# The molecular classification of hereditary endocrine diseases

**DOI:** 10.1007/s12020-015-0674-y

**Published:** 2015-07-07

**Authors:** Lei Ye, Guang Ning

**Affiliations:** Shanghai Key Laboratory for Endocrine Tumors, Shanghai Clinical Center for Endocrine and Metabolic Diseases, Shanghai Institute of Endocrine and Metabolic Diseases, Ruijin Hospital, Shanghai Jiaotong University, School of Medicine, 197 Ruijin 2nd Road, Shanghai, 200025 People’s Republic of China; Laboratory for Endocrine & Metabolic Diseases, Institute of Health Science, Shanghai Jiaotong University School of Medicine and Shanghai Institutes for Biological Sciences, Chinese Academy of Sciences, 227 South Chongqing Road, Shanghai, 200025 People’s Republic of China

**Keywords:** Hereditary endocrine diseases, Genotyping, Molecular classification

## Abstract

Hereditary endocrine diseases are an important group of diseases with great heterogeneity. The current classification for hereditary endocrine disease is mostly based upon anatomy, which is helpful for pathophysiological interpretation, but does not address the pathogenic variability associated with different underlying genetic causes. Identification of an endocrinopathy-associated genetic alteration provides evidence for differential diagnosis, discovery of non-classical disease, and the potential for earlier diagnosis and targeted therapy. Molecular diagnosis should be routinely applied when managing patients with suspicion of hereditary disease. To enhance the accurate diagnosis and treatment of patients with hereditary endocrine diseases, we propose categorization of endocrine diseases into three groups based upon the function of the mutant gene: cell differentiation, hormone synthesis and action, and tumorigenesis. Each category was further grouped according to the specific gene function. We believe that this format would facilitate practice of precision medicine in the field of hereditary endocrine diseases.

## Introduction

Since Vaughan Pendred first described Pendred Syndrome in 1896 [1], there have been approximately 200 hereditary endocrine diseases recorded in OMIM database. A recently published consensus of rare endocrine–metabolic diseases (REMD) [2] identified a disease taxonomy that comprised a total of 166 main disorders, which grew to 338 when all variants and subtypes were included. Because of this great heterogeneity, accurate diagnosis remains a challenge for endocrinologists. Importantly, ~90 % of described endocrine syndromes have a defined underlying genetic cause that would allow for definitive diagnosis. Currently, diagnosis is largely based on an anatomical classification, where the specific endocrine gland(s) or involved cell type serve as primary route to disease identification [3]. Unfortunately, anatomical location alone only partially addresses the pathophysiological mechanism of the disease. The lack of a molecular picture clearly limits a comprehensive understanding of physiological pathogenesis and sometimes leads to errors in diagnosis, as well as, suboptimal treatment. An expanding literature continues to define clear genotype/phenotype correlations, which work to efficiently guide clinical diagnosis, treatment, and prognosis. For hereditary endocrine diseases that clinically show overlap (e.g., mesenchymal tumor in MEN1 and TSC), uncovering the unique genetic basis provides absolute clarity in the differential diagnosis [3]. The other obvious advantage of genetic testing is the opportunity for uncovering multiple sites involvement in syndromic disorders. For example, hyperparathyroidism with germline MEN1 mutation obligates screening of pituitary, pancreas, and gastrointestinal tract for potential tumorigenesis [4]. Numerous additional examples exist where genetic diagnosis plays a key role in guiding diagnosis and appropriate treatment. Thus, we strongly believe that the current anatomical classification should be supplemented with an approach that includes molecular genotyping.

Currently, over 300 genes have been identified as targets for causal mutations in hereditary endocrine diseases. Advances in DNA sequencing have continued to expand the breadth of genes at an ever-reducing cost. We are quickly approaching a point where molecular diagnosis should be routinely applied when managing patients with hereditary disease. As a result, the use of molecular classification needs to be reconsidered. In fact, the concept of using molecular classification for hereditary endocrine disease was proposed a decade ago by Prof. Marx [3]. The purpose of this manuscript is to describe a molecular classification to serve as basis for classification of hereditary endocrine disease. The molecular classification is based upon the biological function of the mutated genes. As shown in Table 1, we recommend three broad categories: cell differentiation, hormone synthesis/action, and tumoriogenesis. A discussion of the rationale for the three categories and examples of genes that would fall into each group follows.

**Table 1:** Molecular classification of hereditary endocrine diseases

Category	Sub-category	e.g.	Associated diseases	Tissue
Cell differentiation	Genes encoding transcription factor	SRY, DAX1	AHC&HH	Adrenal and pituitary
Hormone synthesis and action	Genes encoding peptide hormone	AVP	ADNDI	Posterior pituitary
	Genes encoding hormone synthesis enzyme	CYP21A2	CAH	Adrenal
	Genes encoding transporter for synthetic materials	PDS	Pendred syndrome	Thyroid
	Genes encoding membrane receptor	INR	Insulin resistance/hypersensitivity	All tissues
	Genes encoding nuclear receptor	GR	Glucocorticoid resistance/hypersensitivity	All tissues
	Genes encoding post-receptor signal molecule	GNAS1	McCune-Albright syndrome/PHP1A/PPHP/POH	Bone/parathyroid and etc.
	Genes encoding ion channel	SCN4A	Liddle syndrome	Kidney
Tumoriogenesis	Oncogene	RET	MEN2	Thyroid C cell/adrenal medulla/parathyroid and etc
	Tumor suppressor gene	MEN1	MEN1	Parathyroid/islet/pituitary and etc.

## Category of cell differentiation

Specific cell lineage development and differentiation is the first step in constructing a functional endocrine system. This process involves complex spatiotemporal regulation of cell lineage–specific transcription factors expressed in pluripotential stem cells. A genetic abnormality involving any of these key transcription factors is an important cause of congenital dysgenesis or agenesis. Specific examples of dysgenesis exist for all major endocrine glands including pituitary, thyroid, pancreas, adrenals, and gonads. It is also clear that multiple genes may have similar effects on a single gland; for example, loss of PAX8, FOXE1, and NKX2-1 is associated with thyroid gland dysgenesis [5]. Additionally, single gene defects may impact multiple glands. DAX1 is essential for both adrenal and gonad development. Mutation in DAX1 led to adrenal hypoplasia congenita (AHC) and hypogonadotropic hypogonadism (HH) [6]. Currently, treatment for this category of disease is hormone replacement in the lifetime. In the future, generation of functional endocrine gland from embryonic stem cells may be new avenue [7].

## Category of hormone synthesis/action

The two broad classes of hormones include peptide hormones and non-peptide hormones. Peptide hormones are directly translated from an encoding gene and therefore venerable to direct mutation. Genetic defects in an encoding gene (e.g., AVP) typically lead to complete loss of function, impaired function, or failures in post-translation processing of peptide hormone [8]. Mutation of genes in this category usually leads to solitary hormone deficiency (e.g., autosomal dominant neurohypophyseal diabetes insipidus) [8].

Non-peptide hormones are synthesized by a series of synthetic enzymes from natural materials. Therefore, mutation targeting is indirect. Steroid hormones represent the single most important group of non-peptide hormones. They are all derived from cholesterol and therefore share a complex synthetic pathway. Through the concerted action of several enzymes, such as CYP21A2, CYP17A1, CYP11B1, HSD3B2, etc., multiple steroid hormones are produced, degraded, and recycled. For this reason, mutations targeting these genes often have complex phenotypic outcomes, such as congenital adrenal hyperplasia (CAH) [9]. Importantly, mutations targeting early steps of the synthetic pathway effect production of multiple steroids.

In addition to synthesis, the appropriate transportation of synthetic materials is indispensable for non-peptide hormones as well. This gene class when targeted by mutation may lead to impaired hormone synthesis/function. SLC26A4, for example, encodes an iodide chloride transporter that resides in thyroid follicular cells, known as pendrin. Mutated pendrin is retained in the endoplasmic reticulum instead of being trafficked to plasma membrane, therefore decreasing iodide transport, causing hypothyroidism in Pendred syndrome [10].

To respond to a hormone, a target cell must contain two essential components of a signaling pathway: a function receptor and effector. Hormone receptors mainly include two groups, membrane receptor and nuclear receptor. Inactivating mutations in hormone receptors either cause hormone resistance phenotypically or mimic hormone insufficiency, while activated mutations cause hormone hypersensitivity. Insulin receptor (INSR) and glucocorticoid receptor (GR) are ubiquitously expressed membrane and nuclear receptor, respectively. Activating mutations cause familial hyperinsulinemic hypoglycemia [11] and glucocorticoid hypersensitivity [12], while inactivating mutations cause insulin resistant syndrome [13] and glucocorticoid resistance, respectively [14].

Effectors mainly include signaling molecules and sometimes ion channels as the terminal effector. Post-receptor signaling molecules are mostly downstream of cell membrane receptor and serve a critical role in the intracellular messaging. A well-characterized example involves G protein–coupled receptors (GPCR). GPCRs transduce extracellular signals to hormone-sensitive adenylate cyclase, the activity of which is regulated by at least 2 G proteins, 1 stimulatory (G_s_), and 1 inhibitory (G_i_) [15]. Gs-α is encoded by GNAS and activating mutations of GNAS gene result in constitutively active GPCR and clinically McCune-Albright syndrome, polyostotic fibrous dysplasia, and various endocrine tumors [16]. Inactivating loss-of-function mutations, on the other hand, result in loss of function of GPCR and clinically pseudohypoparathyroidism Ia (PHP1A), pseudopseudohypoparathyroidism (PPHP), and progressive osseous heteroplasia (POH) [17].

Ion channels are important effectors in mediating mineralocorticoids function. Integrated synthesis and function of ion channels are indispensable in maintaining salt homeostasis. Mutations in either the beta subunit (SCNN1B) or the gamma subunit (SCNN1G) cause constitutive activation of the renal epithelial sodium channel (ENaC) [18]. Clinically, patients with these mutations present with inappropriate K^+^ excretion and hypokalemia (Liddle syndrome) [18].

Diseases targeting hormone function may clinically present as either gain or loss of function. For this reason, we include specific sub-categories related to the molecular functions. The categorization of a specific genetic defect occurring in the hormone pathway helps guide optimal treatment. For example, identification of a defective signaling pathway rather than lack of hormone triggers a completely different treatment route, as supplementation becomes a less viable option.

## Category of tumoriogenesis

Tumor syndromes are described for all major endocrine glands, and like other cancers have been linked to activating mutations of oncogenes or inactivating mutations of tumor suppressor genes. Perhaps the best-described examples are activating mutations of RET proto-oncogene in MEN2 [19] and inactivating mutations of MEN1 tumor suppressor gene in MEN1 [20]. Germline RET mutations cause constitutive activation of the RET kinase and downstream signaling pathway, therefore leading to uncontrolled cell proliferation. Targeting RET has opened a new treatment practice in advanced medullary thyroid carcinoma [21]. On the other hand, the germline inactivation of MEN1 requires a ‘second hit’ of the remaining wild type allele to initiate tumorigenesis. While this risk exists in all tissues, the clinical impact is greatest in the parathyroid, pituitary, and pancreas. Tumoriogenesis is a category where genetic testing has a proven benefit that extends well beyond endocrine syndromes. Knowledge of specific mutation status guides treatment of asymptomatic endocrine glands in patients and their relatives before they anatomically identified. Where cancer has progressed, identification of mutational drivers provides the opportunity for targeted therapy.

The benefits derived from defining a molecular classification should be integrated with clinical diagnosis. Accurate clinical diagnosis including defining the specific hormone disorder and localizing the disease lesion is a fundamentally important component of clinical care. Genetic diagnosis provides a means of definitive diagnosis, thereby outlining an accurate treatment strategy for the patient and family members potentially at risk. We are now in a period of transition when genetics is creating the opportunity for a new level of personalized medicine. Recently, President Obama launched the project of Precision Medicine Initiative [22, 23]. While significant advances in precision medicine have been made for select cancers [24], the practice is not currently in use for most diseases, including hereditary endocrine disease. The concept of precision medicine should start with known candidate genes. We believe that as genetic analysis becomes more recognized for its role in patient management, there is value in stratifying mutations by molecular category.
